# Residual HIV activity and host immunometabolic remodeling during antiretroviral therapy: implications for cardiovascular–kidney–metabolic risk

**DOI:** 10.3389/fimmu.2026.1912904

**Published:** 2026-08-25

**Authors:** Jiahui Zhang, Xuanlan Li, Chen Chen, Zhuoyan Wu, Fengping Qin, Yuxuan Li, Yun Zhang, Chuanyi Ning

**Affiliations:** 1 School of Nursing, Guangxi Medical University, Nanning, China; 2 Education Evaluation and Faculty Development Centre, Guangxi Medical University, Nanning, China; 3 Guangxi Key Laboratory of AIDS Prevention and Treatment, Nanning, China

**Keywords:** antiretroviral therapy, cardiovascular–kidney–metabolic syndrome, HIV, immune activation, immunometabolic remodeling, low-level viremia, residual HIV activity, viral immunology

## Abstract

Antiretroviral therapy (ART) suppresses productive HIV replication but does not eliminate viral persistence, residual transcription, or host immune perturbation. Low-level viremia (LLV), generally referring to detectable plasma HIV RNA below the threshold for virologic failure, has traditionally been interpreted in relation to assay variability, incomplete ART exposure, resistance, or impending rebound. Persistent or recurrent LLV may signal residual HIV activity or residual antigenic exposure, but it is not a direct measure of these processes and should not be equated with ongoing productive replication. Residual viral and antigenic signals may interact with microbial, metabolic, behavioral, adiposity-related, and treatment-related inputs, potentially contributing to trained-immunity-like programs, T-cell dysfunction, mitochondrial dysfunction, redox imbalance, impaired lipid handling, and broader immunometabolic perturbation. We therefore position persistent or recurrent LLV as a pattern-dependent virologic phenotype to be interpreted alongside direct measures of residual HIV activity, host immunometabolic phenotypes, contextual determinants, and established cardiovascular–kidney–metabolic (CKM) risk factors. The CKM framework organizes interrelated metabolic, vascular, and kidney-related vulnerability; it does not define an HIV-specific staging system or position LLV as a cause or staging criterion for CKM syndrome. Future studies are needed to define biologically informative LLV patterns, establish the temporal relationships between accompanying host signatures and CKM-related outcomes, and determine whether such patterns and signatures provide clinically meaningful information beyond established treatment-related and CKM risk factors.

## Highlights

- LLV is a clinical virologic observation rather than a single mechanistic state. Persistent or recurrent LLV may be more biologically informative than isolated viral blips, but LLV should not be equated with ongoing productive replication or residual HIV activity itself.
- Residual HIV activity extends beyond plasma LLV. It may include residual transcription, viral RNA or protein expression, infected-cell clonal expansion, and tissue-associated persistence; these processes may coexist with residual antigenic exposure during ART.
- Persistent immune and immunometabolic perturbations may remain despite virologic suppression. Conventional immune recovery markers, including CD4^+^ T-cell count and the CD4^+^/CD8^+^ ratio, may not fully capture residual immune activation, checkpoint-associated dysfunction, immunosenescence, or metabolic–epigenetic inflammatory memory.
- The CKM framework provides a systems-level structure for organizing metabolic, vascular, and kidney-related vulnerability. These domains are interpreted within interacting viral, microbial, behavioral, adiposity-related, and treatment-related contexts, without redefining CKM staging or positioning LLV as causal.
- The LLV–immunometabolic–CKM framework remains hypothesis-generating and translational. Longitudinal and mechanistic studies should determine which LLV patterns correspond to biologically active residual HIV expression, and whether accompanying host signatures provide clinically meaningful information beyond established treatment-related and CKM risk factors.

## 1 Introduction

Antiretroviral therapy (ART) has transformed HIV infection into a chronic, treatable condition by durably suppressing productive viral replication and reducing AIDS-related mortality (1, 2). Yet ART does not eradicate HIV reservoirs or fully abolish residual viral transcription, viral protein expression, or host immune perturbation (3). In some people living with HIV (PLWH), low-grade inflammation, immune activation, and immunometabolic abnormalities persist despite sustained virologic suppression, indicating that plasma viral suppression does not necessarily equate to complete biological resolution (4, 5). These observations raise a key host–pathogen question: how residual HIV activity interacts with host and contextual determinants to shape long-term tissue vulnerability during ART.

Low-level viremia (LLV), generally defined as detectable HIV RNA below the threshold for virologic failure, provides a clinically measurable entry point into this question. In routine HIV care, LLV has traditionally been interpreted in relation to assay variability, incomplete ART exposure, resistance, or impending rebound. Emerging evidence suggests that persistent or recurrent LLV may also occur in the context of residual HIV activity arising from proviral transcription, clonal expansion of infected cells, or tissue-associated viral expression, and may be accompanied by intermittent antigenic exposure (6, 7). Importantly, LLV should not be equated with continuous productive replication. Its biological interpretation depends on viral load magnitude, persistence, recurrence, sequence dynamics, and the extent to which detectable viral signals are accompanied by host immune activation.

To clarify the current state of knowledge, several evidence boundaries should be made explicit. First, the persistence of HIV reservoirs, residual transcription, and chronic immune perturbation during ART is well established (8). Second, associations of LLV with virologic failure, antiretroviral resistance, inflammation, immune activation, or incomplete immune recovery have been reported, but vary according to LLV definition, viral-load magnitude, sampling intensity, ART exposure, treatment history and adherence (9). Interpretation of accompanying host phenotypes additionally requires consideration of adiposity, comorbidities, and behavioral context. Third, the temporal and mechanistic relationships among LLV, residual HIV activity, residual antigenic exposure, host immunometabolic phenotypes, and cardiovascular–kidney–metabolic (CKM)-related vulnerability remain uncertain and hypothesis-generating rather than established as causal (9, 10).

LLV has been studied mainly as a predictor of virologic failure and antiretroviral resistance (11), whereas chronic comorbidities in PLWH have often been examined through individual organs or isolated clinical outcomes (12, 13). This separation can obscure interactions among virologic, immune, metabolic, treatment-related, and behavioral factors across organ systems. To organize these interrelated risks clinically, we draw on the CKM framework introduced by the American Heart Association in 2023 and subsequently translated into a clinical-practice framework in the 2026 multisociety CKM guideline. This framework organizes metabolic risk factors, kidney dysfunction, and cardiovascular disease across a staged continuum rather than as isolated entities (14, 15). When applied to PLWH, it provides a clinical reference structure within which residual HIV activity and host immunometabolic phenotypes can be considered alongside ART effects, aging, adiposity, comorbidities, and behavioral determinants, without redefining CKM syndrome or assuming that LLV drives CKM progression (16, 17).

This review makes three conceptual contributions. First, it reframes persistent or recurrent LLV as a pattern-dependent clinical signal that may reflect residual HIV activity in selected contexts, rather than as a uniform viral-load category or direct evidence of ongoing productive replication (7, 9). Second, it distinguishes residual HIV activity, residual antigenic exposure, host immunometabolic phenotypes, and contextual determinants while examining how their potentially convergent pathways may shape tissue vulnerability. Third, it uses the CKM framework as a systems-level translational structure to organize metabolic, vascular, and kidney-related trajectories and frame testable questions about the clinical relevance of LLV patterns and accompanying host signatures. This framework remains hypothesis-generating and defines priorities for longitudinal, mechanistic, and interventional validation.

## 2 Defining LLV, viral blips, and residual HIV activity during ART

Definitions of LLV vary across clinical guidelines and research settings because thresholds for viral suppression, assay detection limits, and criteria for virologic failure differ across clinical and public health contexts (18). For population-level monitoring, the World Health Organization generally uses HIV RNA ≤1000 copies/mL as the threshold for virologic suppression (19), whereas the U.S. Department of Health and Human Services defines LLV as confirmed HIV RNA above the lower limit of detection but <200 copies/mL (20). The European AIDS Clinical Society considers HIV RNA >50 and <200 copies/mL to represent a low-level viremic state that warrants repeat testing and close monitoring (21). These thresholds are operational rather than mechanistic and should not be treated as interchangeable definitions of a single biological state (9). Accordingly, LLV should be interpreted according to viral-load magnitude, persistence, recurrence, assay sensitivity, and temporal pattern, rather than on the basis of a single measurement (22).

For the purpose of this review, LLV refers primarily to confirmed detectable plasma HIV RNA above the assay lower limit of detection but below 200 copies/mL during ART (20). Detectable HIV RNA below the assay lower limit of quantification is considered separately as very-low-level or residual viremia (23), whereas higher thresholds used in individual studies or public-health guidelines are treated as study-specific operational categories rather than biologically equivalent states (22). Because LLV-related terminology is used inconsistently across guidelines and studies, **Table 1** defines the working terms used in this review and distinguishes clinical viral-load categories from broader biological concepts of residual HIV activity and residual antigenic exposure.

**Table 1:** Working definitions and conceptual boundaries of LLV-related terms used in this review.

Term	Definition in this review	Conceptual and interpretive boundary	Supporting references
Low-level viremia	Confirmed detectable plasma HIV RNA above the assay lower limit of detection but below 200 copies/mL during ART, unless a different study-specific threshold is explicitly stated.	Detectable HIV RNA below the lower limit of quantification is considered very-low-level or residual viremia rather than being assumed to represent the same biological state as quantifiable LLV.	(19–21)
Viral blip	A transient and isolated detectable HIV RNA measurement after prior virologic suppression, followed by return to virologic suppression on subsequent testing.	A blip should be distinguished from persistent or recurrent LLV and does not necessarily imply sustained viral replication.	(11, 20)
Persistent LLV	Sustained low-level detectable plasma HIV RNA on repeated measurements during ART.	Persistent LLV is more likely than an isolated blip to raise concern for repeated viral output, incomplete drug exposure, resistance risk, or reservoir-derived activity, although mechanisms may differ across individuals.	(9, 11, 13, 24)
Recurrent or intermittent LLV episodes	Repeated episodes of low-level detectable plasma HIV RNA over time, with intervening periods of virologic resuppression or HIV RNA below the assay limit of quantification or detection.	This term is used descriptively rather than as a universally standardized guideline category and should be evaluated longitudinally rather than from a single measurement.	(25–27)
Residual HIV activity	Ongoing or intermittent HIV-related biological processes during ART, including residual transcription, viral RNA or protein expression, infected-cell clonal expansion, tissue-associated reservoir activity, or release of viral products.	Residual HIV activity is broader than plasma LLV. It may occur without measurable LLV, and LLV does not necessarily indicate ongoing cycles of productive replication.	(6, 7, 28, 29)
Residual antigenic exposure	Ongoing or intermittent exposure of host immune cells to HIV-derived antigenic or immunostimulatory products, including viral proteins, RNA, viral particles, infected-cell products, or tissue-associated viral signals.	Residual antigenic exposure is a potential biological bridge between residual HIV activity and persistent immune activation, but it is not synonymous with LLV, virologic failure, or productive replication.	(28, 30, 31)

Persistent or recurrent LLV may serve as a clinically measurable signal of biologically relevant residual HIV activity in selected contexts, but it is not synonymous with residual HIV activity or ongoing productive replication. Among PLWH receiving ART, LLV is relatively common, with reported prevalence estimates of approximately 10%–30% (13). Estimates vary across cohorts and care settings because viral-load monitoring intervals, assay detection limits, adherence patterns, ART regimen potency, treatment history, and access to resistance testing differ substantially. Clinically, detectable low-level HIV RNA may appear as transient blips, intermittent episodes, or persistent or recurrent LLV (24). These longitudinal patterns are more informative than a single detectable result because they may reflect different processes, including assay-level variation, transient biological fluctuation, incomplete drug exposure, release of viral RNA from infected-cell clones, intermittent proviral transcription, or tissue-associated viral expression (32).

The clinical significance of LLV has traditionally been evaluated in relation to subsequent virologic failure and the emergence of antiretroviral resistance (9, 11, 33). A viral blip is an isolated detectable HIV RNA measurement after prior virologic suppression that is followed by return to virologic suppression on subsequent testing; it can therefore be confirmed only retrospectively after resuppression has been documented (20). Possible explanations include assay variability, transient biological fluctuation, temporary release of viral RNA from infected cells or expanded cellular clones, short-lived changes in adherence or effective drug exposure, and intercurrent immune activation. An isolated low-amplitude blip does not usually predict virologic failure and should not, by itself, prompt modification of an otherwise effective ART regimen (20, 21).

In contrast, higher-magnitude blips, repeated detectable measurements, or failure to resuppress may indicate evolving persistent or recurrent LLV, incomplete ART exposure, emerging resistance, or early loss of virologic control. Persistence, recurrence, magnitude, and longitudinal trajectory should therefore guide interpretation rather than the mere occurrence of one detectable result.

Clinical assessment should begin with repeat HIV RNA testing to distinguish an isolated blip from recurrent LLV, persistent LLV, and virologic rebound (9). Evaluation should also include adherence, treatment interruptions, drug–drug and drug–food interactions, impaired absorption, regimen history, and resistance history (21). Confirmed LLV below 200 copies/mL does not generally require automatic modification of an otherwise effective regimen, but continued virologic monitoring is warranted, generally at least every three months (20). Confirmed HIV RNA ≥ 200 copies/mL should instead prompt evaluation for virologic failure and, where feasible, resistance testing. No LLV threshold has been validated to predict inflammation or tissue injury at the individual-patient level; inflammatory or CKM-related biomarkers should therefore complement, rather than replace, longitudinal virologic assessment.

Beyond virologic outcomes, LLV may also have broader clinical and pathobiological relevance (34). Several studies have associated LLV with elevated inflammatory markers, increased T-cell activation, immune senescence, incomplete CD4^+^ T-cell recovery, and persistently low CD4^+^/CD8^+^ ratios (35, 36). These associations vary across LLV definitions, populations, treatment contexts, and measurement strategies and do not establish that LLV directly causes immune or tissue injury (35). Persistent or recurrent LLV may nevertheless identify a subset of treated PLWH with incomplete immune resolution or accompanying residual antigenic exposure, whereas isolated low-level detections should not be assumed to have equivalent biological significance. The following sections examine the viral sources and associated host-response pathways that may contribute to, or accompany, these associations.

## 3 Virologic sources of residual HIV expression under ART

### 3.1 Intact and defective proviruses as sources of residual viral RNA and proteins

The persistent HIV reservoir is composed predominantly of infected cells harboring integrated proviruses, including replication-competent intact proviruses and a larger population of defective proviruses (37, 38). Intact proviruses can generate infectious virions after cellular activation and remain a major source of viral rebound after ART interruption (39). Although defective proviruses cannot complete a full replication cycle, a subset may retain transcriptional or translational capacity and produce viral RNA or proteins (6, 40). These observations indicate that residual HIV activity under ART does not necessarily require continuous cycles of productive replication. Even limited production of viral RNA or proteins may provide immunostimulatory or antigenic signals to sustain immune recognition, particularly when such output is repeated, persistent, or compartmentalized.

### 3.2 Clonally expanded infected cells and low-level viral output

Molecular studies have shown that viral sequences detected during LLV are often highly homogeneous and show limited evidence of ongoing viral evolution, supporting a contribution from clonally expanded infected cells (8). These infected CD4^+^ T-cell clones can persist through homeostatic proliferation, antigen-driven expansion, or integration site-associated selective proliferation (41, 42). When intermittently activated or transcriptionally permissive, such clones may release residual viral RNA, express viral proteins, or generate low-level particles without necessarily indicating widespread cycles of replication (43). In this context, LLV may represent a measurable signal of reservoir-derived viral output or clonal persistence, rather than simply an unstable treatment response.

Nevertheless, clonal expansion does not explain all forms of LLV. Detectable low-level HIV RNA may arise through different processes across individuals, anatomical compartments, ART histories, immune activation states, and testing contexts (44). LLV should therefore be understood as a heterogeneous virologic phenotype with multiple potential sources rather than as a single mechanistic entity. This heterogeneity is central to its biological interpretation: some LLV patterns may reflect assay variation or incomplete drug exposure, whereas others may be consistent with residual viral expression capable of sustaining antigenic exposure.

### 3.3 Tissue-associated reservoirs and compartmentalized antigenic exposure

The HIV reservoir is not confined to resting CD4^+^ T cells in peripheral blood. Viral persistence also occurs within tissue microenvironments that differ in immune composition, drug penetration, inflammatory tone, and metabolic activity (45, 46). These niches may support local proviral transcription and residual antigen production even when plasma HIV RNA is suppressed or only intermittently detectable. Tissue-associated reservoirs may therefore be particularly relevant as sources of compartmentalized antigenic exposure, although their quantitative contribution to measurable plasma LLV remains uncertain.

Adipose tissue represents one such immunometabolic niche. It contains CD4^+^ T cells, macrophages, stromal cells, and other immune cell subsets and functions as both an endocrine and immune-regulatory organ (47). Within this microenvironment, residual viral expression may intersect with macrophage activation, adipokine dysregulation, and local metabolic stress (48). Although the extent to which adipose tissue contributes directly to plasma LLV remains unclear, this niche may provide a biologically plausible link between HIV persistence, local inflammation, and metabolic dysfunction.

Gut-associated lymphoid tissue is another important site of HIV persistence. It is enriched in CD4^+^ T cells and remains continuously exposed to microbial and dietary antigens (39, 49). Incomplete mucosal immune restoration after ART may sustain local inflammation and barrier dysfunction, creating conditions that favor both viral persistence and immune activation (50). In this setting, microbial translocation should be viewed as a parallel inflammatory amplifier rather than a direct source of LLV.

These peripheral tissue niches may sustain local viral or antigenic activity and shape systemic inflammatory and metabolic phenotypes, although their quantitative contribution to measurable plasma LLV remains uncertain. The central nervous system (CNS), however, represents a biologically distinct anatomical and pharmacological compartment in which viral persistence is more appropriately evaluated through brain tissue or cerebrospinal fluid than inferred from plasma HIV RNA alone. HIV may persist within the CNS during ART, including in brain-resident microglia and other susceptible cellular populations (51). Persistent HIV transcription has been demonstrated in brain tissue from virally suppressed PLWH, and inducible replication-competent virus has been recovered from brain-derived microglia in ART-treated individuals (45, 52). Compartmentalized CNS viral activity may also become manifest as cerebrospinal fluid (CSF) HIV RNA escape, in which HIV RNA is detectable or higher in CSF despite suppressed or lower plasma HIV RNA (53).

These observations do not establish the CNS as a routine source of measurable plasma LLV; low-level CSF HIV RNA and CSF viral escape should therefore be distinguished from peripheral plasma LLV. Within the CNS, persistent viral or antigenic signals may coexist with microglial activation, neuroinflammation, blood–brain barrier dysfunction, and oxidative or mitochondrial stress (51). Experimental studies further suggest that HIV proteins such as Tat can disrupt microglial lipid and cholesterol metabolism and promote lipid-droplet accumulation and inflammatory activation (54). Such processes may contribute to axonal injury or neurocognitive symptoms in selected individuals, although the long-term consequences of asymptomatic or very-low-level CSF HIV RNA remain incompletely defined (51, 53).

These CNS-specific observations illustrate compartmentalized host remodeling rather than a confirmed pathway linking plasma LLV to neurological injury. Although CNS vulnerability may share inflammatory, metabolic, and vascular determinants with CKM-related risk, neurological outcomes are not part of formal CKM staging (15), and a direct pathway from CNS HIV persistence to CKM progression has not been established.

### 3.4 Epigenetic regulation of latency and intermittent transcription

The persistence and transcriptional activity of the HIV reservoir are shaped in part by the host epigenetic landscape. During infection, HIV integration and proviral activity are influenced by chromatin accessibility, histone modifications, and local transcriptional programs, including activating marks such as H3K4me3 and H3K27ac (8). Under long-term ART and immune selection pressure, reservoir composition may gradually shift, with a subset of intact proviruses becoming enriched in transcriptionally repressive genomic regions that favor latency and immune evasion (55). However, latency does not necessarily imply complete transcriptional silence. Some proviruses may undergo intermittent or low-level transcription and produce viral RNA or proteins despite effective ART (28, 56).

Such residual transcription may be insufficient to cause overt virologic failure but may still contribute to immune stimulation, particularly when accompanied by translation or release of viral products. In selected contexts, epigenetically regulated intermittent transcription may also contribute to detectable LLV. These mechanisms link latent HIV persistence to host immune engagement by showing how quantitatively limited viral output may become biologically meaningful when it is recurrent, compartmentalized, or embedded within inflammatory tissue microenvironments.

## 4 Host antiviral sensing of residual HIV signals and durable immune activation

### 4.1 Innate sensing and trained-immunity-like reprogramming

Repeated exposure to HIV-derived molecular patterns and host-derived danger signals may recalibrate innate immune responses in monocytes and macrophages and, in some settings, contribute to durable functional reprogramming consistent with trained immunity (57). Trained-immunity programs are characterized by enhanced responses to subsequent viral antigens, microbial products, or lipid stimuli (58) and are supported by metabolic and epigenetic changes that may sustain heightened inflammatory responsiveness (57, 58).

In treated HIV infection, intermittent or persistent antigenic exposure associated with residual HIV activity could plausibly interact with microbial ligands and other sterile inflammatory stimuli in shaping persistent innate hyper-responsiveness. However, direct evidence linking LLV, as a plasma virologic observation, to trained-immunity-like reprogramming in PLWH remains limited. Such reprogramming should be regarded as a candidate host-response mechanism within a multifactorial inflammatory milieu, rather than as an established downstream consequence of LLV or a confirmed causal link between residual viral sensing and subsequent adipose and vascular remodeling.

### 4.2 Adaptive immune dysfunction and incomplete immune recovery under recurrent antigenic pressure

Recurrent antigenic stimulation may also reshape adaptive immunity. Chronic low-grade antigen exposure can impair CD4^+^ and CD8^+^ T-cell function and promote features of immune exhaustion, including sustained expression of inhibitory immune checkpoint molecules, reduced proliferative capacity, impaired cytotoxic function, and dysregulated cytokine production (37, 59). Persistent inflammation may further damage CD4^+^ T cells through bystander injury and inflammatory cell-death pathways, including pyroptosis in susceptible lymphoid-cell contexts (50). Sustained immune attrition may skew naïve T-cell differentiation toward effector and memory phenotypes, accompanied by expansion of terminally differentiated-like effector memory T cells re-expressing CD45RA (TEMRA cells) (35). Although this remodeling may preserve short-term effector capacity, it can progressively erode the naïve T-cell pool, restrict T-cell receptor repertoire diversity, and accelerate immunosenescence (60).

Importantly, conventional indices of immune recovery may not fully capture the depth or quality of immune restoration during ART. CD4^+^ T-cell count primarily reflects quantitative reconstitution, whereas the CD4^+^/CD8^+^ ratio provides a broader but still incomplete estimate of immune balance. Persistent immune activation, T-cell senescence, and checkpoint-associated exhaustion may remain detectable despite virologic suppression and apparent CD4^+^ T-cell recovery (60–62). Accordingly, immune recovery during ART should be understood not only as numerical restoration of CD4^+^ T cells, but also as partial normalization of immune phenotype, repertoire diversity, proliferative capacity, and effector function (61).

Immune checkpoint pathways are particularly relevant in this context. Increased expression of inhibitory receptors, including programmed cell death protein 1 (PD-1), T-cell immunoglobulin and mucin domain-containing protein 3 (TIM-3/HAVCR2), as well as regulatory ligands such as programmed death-ligand 1 (PD-L1), may reflect persistent antigen exposure and incomplete functional recovery (63, 64). The PD-1/PD-L1 axis can dampen T-cell receptor signaling, limit proliferative responses, and alter cytokine production, thereby contributing to a state in which T cells remain phenotypically activated but functionally constrained (63, 65). These markers may provide complementary information beyond CD4^+^ T-cell count and CD4^+^/CD8^+^ ratio, although their associations with standard immune recovery metrics may be modest and should not be interpreted as definitive surrogate markers without further validation.

Recent cellular immunophenotyping studies suggest that LLV may be accompanied by an unfavorable immune footprint that is not always captured by soluble inflammatory biomarkers. In older adults receiving long-term ART, LLV below 200 copies/mL has been associated with increased T-cell activation and senescence-related phenotypes, including higher expression of PD-1 and TIM-3/HAVCR2, even when systemic inflammatory markers did not clearly differ from those in virologically suppressed individuals (35). These findings suggest that persistent or recurrent LLV may identify a subset of ART-treated PLWH with ongoing T-cell activation, checkpoint-associated dysfunction, and immunosenescence; whether these phenotypes reflect residual antigenic stimulation requires direct assessment (31, 66).

Under recurrent antigenic pressure, these alterations may coexist and potentially reinforce persistent immune activation, checkpoint-mediated T-cell dysfunction, immunosenescence, and incomplete immune recovery. This interpretation offers a potential explanation for why virologic suppression and improvement in conventional CD4^+^ T-cell metrics do not necessarily indicate full restoration of immune competence in all ART-treated PLWH.

### 4.3 Viral protein-mediated cellular stress

Residual viral expression under ART may also generate viral proteins, including Tat, Nef, gp120, and Vpr, even in the absence of a complete productive replication cycle (31). Experimental studies indicate that these proteins can perturb immune cells, endothelial cells, adipocytes, and renal cells through inflammatory signaling, oxidative stress, endoplasmic reticulum stress, and metabolic stress responses. Vpr has been implicated in pathways involving hypoxia-inducible factor 1α (HIF-1α) and peroxisome proliferator-activated receptor γ (PPAR-γ), potentially promoting glycolytic reprogramming and a pro-inflammatory macrophage phenotype (48). Nef has been linked to impaired ATP-binding cassette transporter A1 (ABCA1)-mediated cholesterol efflux, favoring lipid accumulation and foam-cell formation (67, 68). Tat and gp120 can disrupt calcium homeostasis and protein-folding networks, thereby promoting endoplasmic reticulum stress, oxidative stress, and endothelial injury (69). These findings support biological plausibility but do not establish that persistent or recurrent LLV is consistently accompanied by comparable viral-protein exposure or cellular stress *in vivo*.

### 4.4 Microbial translocation as a parallel amplifier

Microbial translocation is an important amplifier of HIV-associated chronic inflammation (70), but it should be distinguished from the virologic sources of LLV. HIV infection can disrupt intestinal epithelial integrity, deplete mucosal Th17 cells, and impair mucosal immune restoration, allowing microbial products such as lipopolysaccharide and β-D-glucan to enter the systemic circulation (49, 70). These gut-derived ligands can activate monocytes and macrophages through pattern-recognition receptor pathways and promote the release of pro-inflammatory mediators, including IL-6 and TNF-α (49).

Diet quality, fiber intake, and gut microbial ecology may modify the magnitude and inflammatory consequences of microbial translocation through effects on microbial metabolites and epithelial barrier integrity (70–72). In PLWH, Mediterranean-style and high-fiber dietary patterns have been associated with selected microbial, immune, proteomic, and metabolic profiles (73, 74). These observations support considering diet as a hypothesis-generating host-directed pathway that could modulate systemic inflammation by influencing gut microbial ecology, microbial metabolites, mucosal barrier integrity, and microbial translocation. However, interventional studies in ART-treated PLWH remain limited and have primarily assessed microbiome, inflammatory, or metabolic outcomes; whether dietary interventions modify LLV, residual HIV activity, or reservoir measures remains unknown (74, 75).

Residual HIV-derived antigens or proteins may provide intermittent immune stimulation, whereas translocated microbial ligands represent a parallel, non-virologic input into overlapping innate immune pathways (49). Their coexistence could contribute to persistent inflammatory responsiveness, but their relative contributions and temporal relationships remain uncertain (70). Repeated exposure to microbial and other inflammatory stimuli may be compatible with trained-immunity-like reprogramming (76); however, direct evidence that microbial translocation and LLV converge through trained-immunity-like reprogramming in PLWH remains limited.

**Figure 1:**
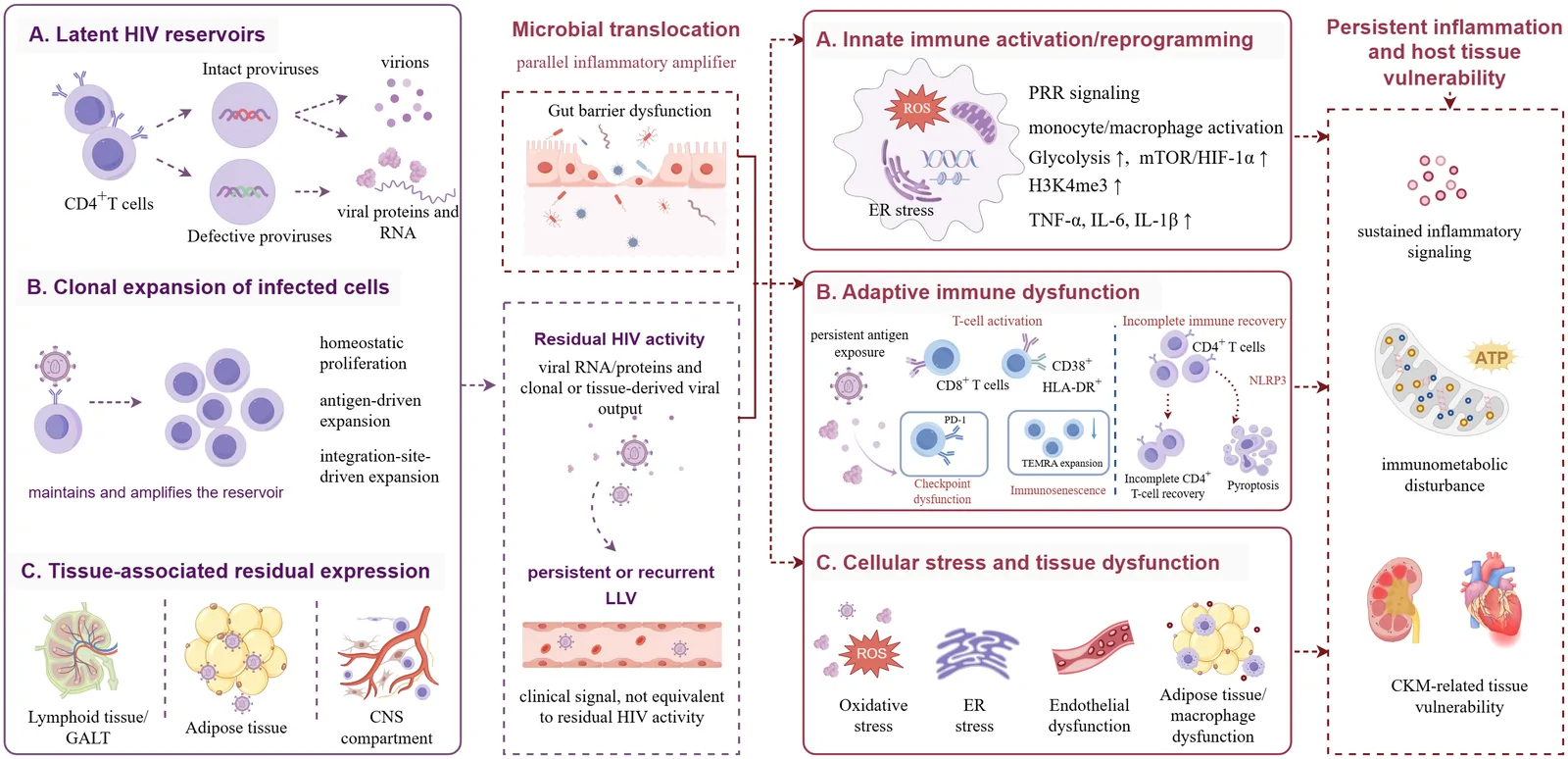
Potential relationships among residual HIV activity, LLV, and host immune perturbation during antiretroviral therapy. Latent intact or defective proviruses, clonal expansion of infected cells, and tissue-associated residual expression may sustain intermittent production of viral RNA, proteins, or virions during ART. These processes constitute residual HIV activity, which may occur with or without measurable plasma LLV; persistent or recurrent LLV is presented as a clinical signal rather than an equivalent measure of residual HIV activity or ongoing productive replication. Microbial translocation is shown as a parallel inflammatory amplifier. Residual viral or antigenic signals and microbial products may converge on innate immune activation, adaptive immune dysfunction, and viral-protein-associated cellular stress, potentially accompanying chronic inflammation, immunometabolic disturbance, and CKM-related tissue vulnerability. Dotted arrows denote proposed or biologically plausible relationships and do not establish a linear causal pathway from LLV to host or organ injury. ER, endoplasmic reticulum; GALT, gut-associated lymphoid tissue; HIF-1α, hypoxia-inducible factor 1 alpha; HLA-DR, human leukocyte antigen-DR; IL, interleukin; mTOR, mechanistic target of rapamycin; NLRP3, NOD-like receptor family pyrin domain-containing 3; PD-1, programmed cell death protein 1; PRR, pattern-recognition receptor; ROS, reactive oxygen species; TEMRA, terminally differentiated effector memory T cells re-expressing CD45RA; TNF-α, tumor necrosis factor alpha. Created by figdraw.com.

Microbial translocation should be regarded as a context-dependent inflammatory amplifier rather than a source of LLV or evidence of a confirmed linear pathway from residual HIV activity to trained immunity and CKM-related vulnerability. These relationships are summarized in **Figure 1**.

## 5 Host immunometabolic remodeling in the context of residual HIV-associated and non-viral stressors

In treated HIV infection, residual antigenic exposure and viral proteins should be understood within a broader inflammatory and metabolic milieu. Together with microbial ligands, selected ART-related metabolic effects, and modifiable behavioral factors, these inputs may converge on shared cellular stress pathways involving redox imbalance, mitochondrial dysfunction, altered energy metabolism, defective lipid handling, and endoplasmic reticulum stress. These pathways are not uniquely attributable to residual HIV activity or LLV; rather, their relevance in treated HIV infection lies in the repeated and potentially interacting engagement of common stress-response networks by viral, antigenic, microbial, metabolic, behavioral, and treatment-related inputs. Over time, such repeated stimulation may shift otherwise adaptive stress responses toward persistent metabolic inflexibility, impaired repair, and heightened sensitivity to secondary metabolic or hemodynamic insults.

### 5.1 Redox imbalance, mitochondrial dysfunction, and inflammasome activation

Excessive reactive oxygen species (ROS) can be generated by activated monocytes, macrophages, and neutrophils (58). HIV proteins, including Tat, Nef, and gp120, may further increase ROS production, partly through NADPH oxidase-dependent pathways (77). Certain ART agents can also contribute to oxidative and metabolic stress through mitochondrial toxicity (48). Sustained ROS overproduction can damage lipids, proteins, and DNA, reduce nitric oxide bioavailability, impair endothelium-dependent vasodilation (50), and promote low-density lipoprotein (LDL) oxidation and foam-cell formation (67).

Mitochondrial injury provides a central link between metabolic stress and inflammatory signaling (78). Viral proteins and chronic inflammatory mediators can impair mitochondrial membrane potential, electron transport chain activity, and ATP production (79). Damaged mitochondria may release mitochondrial DNA, cardiolipin, and other damage-associated molecular patterns, which can activate the NOD-like receptor family pyrin domain-containing 3 (NLRP3) inflammasome and the cyclic GMP–AMP synthase–stimulator of interferon genes (cGAS–STING) pathway, inducing IL-1β, IL-18, and type I interferon-related responses (48, 60, 80, 81). In this way, ROS excess and mitochondrial dysfunction may reinforce one another, sustaining inflammasome activation and inflammatory signaling.

### 5.2 Glycolytic rewiring and metabolic–epigenetic reinforcement of inflammation

Repeated or sustained exposure to viral antigens, microbial ligands, and inflammatory cytokines can reshape immune cell metabolism. In monocytes and macrophages, inflammatory cues can favor a shift from oxidative phosphorylation toward aerobic glycolysis, providing rapid energy and biosynthetic intermediates while sustaining a pro-inflammatory phenotype (57). Consistent with mechanisms described in trained-immunity models, activation of the mechanistic target of rapamycin (mTOR)/HIF-1α axis may further enhance glycolytic flux, inflammatory gene expression, and pro-inflammatory macrophage polarization (58).

This metabolic shift is not merely a consequence of inflammation; it may also reinforce inflammatory memory through epigenetic regulation. Changes in acetyl-CoA, NAD^+^, succinate, and α-ketoglutarate can alter histone acetylation, histone methylation, and chromatin accessibility (57, 79). Through this metabolic–epigenetic coupling, transient inflammatory stimulation may become consolidated into a more durable pro-inflammatory transcriptional state. In T cells, prolonged antigenic stimulation can increase energetic demand and promote a highly glycolytic, bioenergetically stressed phenotype, which may reduce mitochondrial reserve, weaken metabolic adaptability, and contribute to functional exhaustion (35, 59).

### 5.3 Lipotoxicity, endoplasmic reticulum stress, and impaired insulin signaling

Lipid overload is not merely a biochemical feature of dyslipidemia; it can also function as a cellular danger signal, particularly when oxidative and mitochondrial stress impair lipid handling. Intracellular cholesterol accumulation and lipotoxic intermediate formation may result from convergent processes, including Nef-mediated impairment of cholesterol efflux, LDL oxidation, and defective mitochondrial fatty acid β-oxidation (49, 67, 82). Ceramides and diacylglycerols can activate stress kinases such as protein kinase C (PKC) and c-Jun N-terminal kinase (JNK), induce inhibitory phosphorylation of insulin receptor substrate 1 (IRS-1), and impair phosphoinositide 3-kinase/protein kinase B (PI3K/Akt) signaling, thereby disrupting insulin signal transduction (82). By converging with ROS production and NLRP3 inflammasome activation, these lipid-derived stress signals may amplify inflammation while impairing insulin responsiveness.

**Figure 2:**
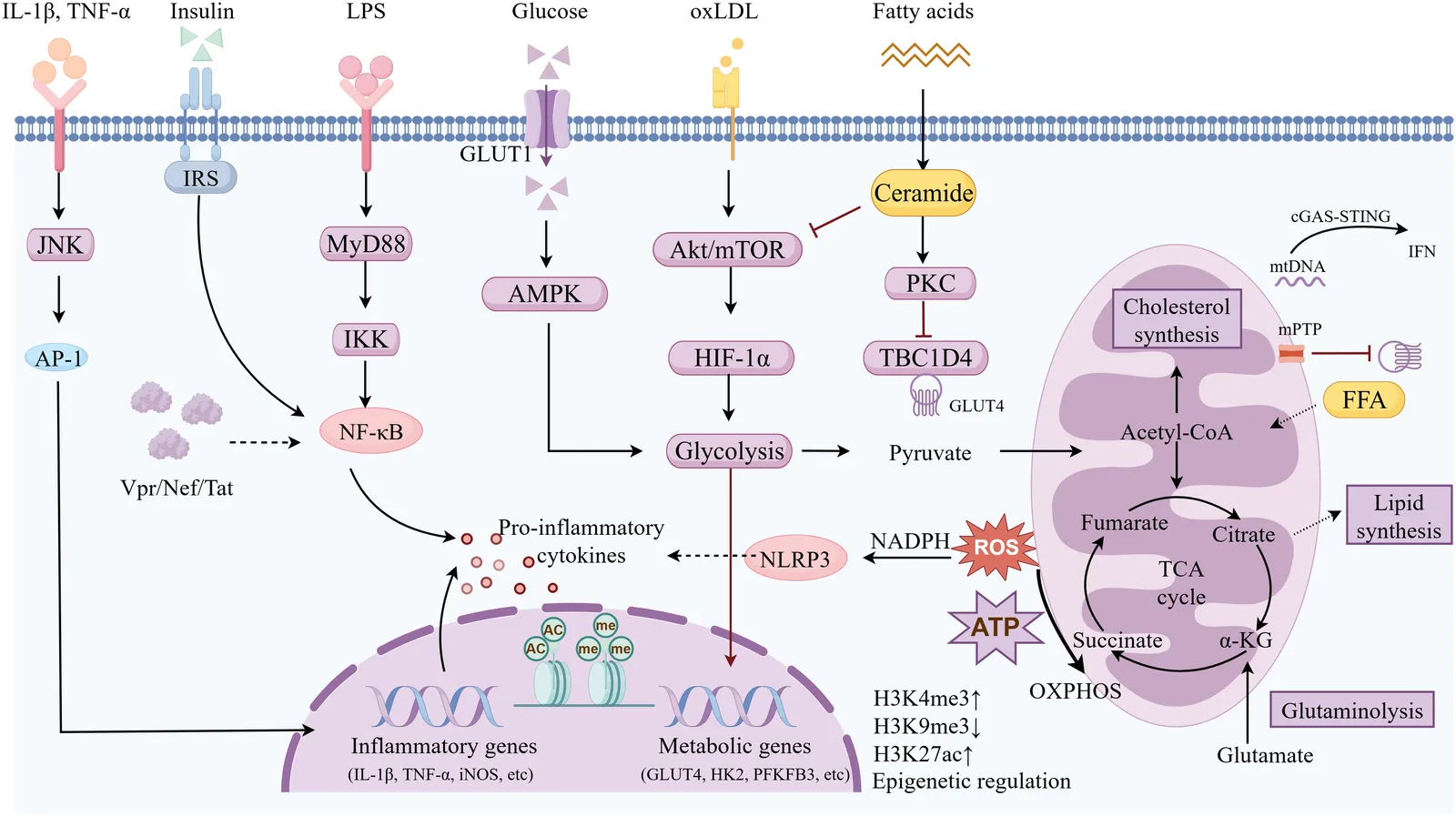
Shared cellular stress-response networks in host immunometabolic remodeling during treated HIV infection. Inflammatory cytokines, microbial products, glucose, insulin, oxidized lipids, fatty acids, and residual HIV proteins are shown as potential inputs into interconnected inflammatory and metabolic pathways. These inputs may influence NF-κB signaling, glucose transport and glycolysis, insulin signaling, lipid and cholesterol synthesis, and mitochondrial substrate utilization. Mitochondrial dysfunction may increase ROS production, impair ATP generation, release mitochondrial DNA, and activate cGAS–STING or NLRP3-related inflammatory signaling. Changes in metabolic intermediates and histone modifications may further support persistent inflammatory or metabolic gene programs. The illustrated pathways represent candidate convergent networks rather than a single sequence occurring uniformly in all cell types, and they are not specific to LLV or residual HIV activity. α-KG, alpha-ketoglutarate; Akt, protein kinase B; AMPK, AMP-activated protein kinase; AP-1, activator protein 1; ATP, adenosine triphosphate; cGAS–STING, cyclic GMP–AMP synthase–stimulator of interferon genes; FFA, free fatty acids; GLUT1 and GLUT4, glucose transporters 1 and 4; HIF-1α, hypoxia-inducible factor 1 alpha; IFN, interferon; IKK, inhibitor of NF-κB kinase; IL-1β, interleukin-1 beta; IRS, insulin receptor substrate; JNK, c-Jun N-terminal kinase; LPS, lipopolysaccharide; mPTP, mitochondrial permeability transition pore; mtDNA, mitochondrial DNA; mTOR, mechanistic target of rapamycin; MyD88, myeloid differentiation primary response 88; NF-κB, nuclear factor-kappa B; NLRP3, NOD-like receptor family pyrin domain-containing 3; OXPHOS, oxidative phosphorylation; oxLDL, oxidized low-density lipoprotein; PKC, protein kinase C; ROS, reactive oxygen species; TCA, tricarboxylic acid cycle. Ac and me indicate histone acetylation and methylation, respectively. Created by figdraw.com.

At the interface of lipid and inflammatory stress, persistent protein-folding stress may activate the unfolded protein response and, if unresolved, promote cytokine production, apoptosis, and profibrotic signaling (69). These interconnected stress programs provide a plausible cellular basis for the metabolic, vascular, and kidney tissue vulnerability discussed below. The shared cellular stress network is illustrated in **Figure 2**.

## 6 CKM-related tissue vulnerability in a multifactorial immunometabolic context

In PLWH, metabolic, vascular, and kidney vulnerability emerges from the convergence of aging, conventional cardiometabolic risk factors, modifiable host and behavioral factors, long-term ART-related effects, chronic inflammation, and residual viral or antigenic stimulation. Likewise, LLV should not be treated as a standalone cause of organ injury. Rather, in selected individuals, it may coexist with residual antigenic exposure or incomplete immune resolution within this broader background of metabolic, inflammatory, hemodynamic, behavioral, and treatment-related stress. Accordingly, the tissue phenotypes discussed below should be understood as convergent vulnerability states rather than organ-specific consequences attributable to LLV or residual HIV activity alone.

### 6.1 Adipose tissue inflammation and impaired metabolic buffering

Adipose tissue is a metabolically active immune niche in treated HIV infection. In the setting of HIV infection and long-term ART exposure, adipose tissue may undergo fat redistribution, immune cell infiltration, adipokine imbalance, and insulin resistance (IR) (47). Chronic immune activation promotes monocyte recruitment, macrophage infiltration, M1-like polarization, and sustained production of inflammatory mediators such as IL-6 and TNF-α (57). Reduced adiponectin and increased pro-inflammatory adipokines may impair the anti-inflammatory and insulin-sensitizing functions of adipose tissue (83, 84). When adipose lipid storage capacity is exceeded or impaired, excess lipids may accumulate in non-adipose organs, promoting lipotoxic stress, impaired insulin signaling, and systemic metabolic dysfunction (48, 82). HIV-related factors and ART-related metabolic effects may further impair adipose tissue buffering through Vpr-related effects on adipocyte precursor differentiation and macrophage metabolism, as well as ART-associated dyslipidemia, weight gain, or fat redistribution (48, 85–87).

### 6.2 Vascular inflammation, endothelial dysfunction, and atherosclerotic remodeling

Inflammatory cytokines such as IL-6 and TNF-α can impair endothelial nitric oxide signaling, reduce nitric oxide bioavailability, and promote endothelial dysfunction (88). Upregulation of adhesion molecules, including ICAM-1, VCAM-1, and MCP-1, promotes monocyte adhesion to the vascular endothelium and migration into the vessel wall (69, 89). Under conditions of oxidative stress and lipid dysregulation, uptake of oxidized LDL by monocyte-derived macrophages can promote foam-cell formation, lipid deposition, and atherosclerotic plaque progression (50, 67, 90). Mechanisms consistent with trained-immunity-like reprogramming may contribute to persistent pro-inflammatory monocyte and macrophage phenotypes and thereby intensify local plaque inflammation (91, 92). Viral-protein-associated oxidative stress, endoplasmic reticulum stress, and lipid stress may further intersect with these vascular pathways (67, 77). In individuals with persistent or recurrent LLV, residual antigenic exposure may coexist with sustained immune activation and vascular lipid stress, potentially adding to established atherosclerotic risk pathways; however, the independent contribution of LLV remains uncertain (93).

### 6.3 Kidney vulnerability to metabolic, inflammatory, and hemodynamic stress

The kidney is particularly susceptible to immunometabolic stress because of its high perfusion rate, high energy demand, and continuous exposure to hemodynamic and toxic insults (94). IR, hypertension, and endothelial dysfunction can increase renal hemodynamic stress, contributing to altered vasomotor tone, glomerular hypertension, and hyperfiltration (95, 96). Glomerular hypertension and hyperfiltration can damage podocytes and the filtration barrier, increase the risk of albuminuria, and promote glomerulosclerosis (95, 97). In parallel, adipose tissue dysfunction can increase circulating free fatty acids and reduce adiponectin, weakening renal antioxidant, anti-apoptotic, and metabolic protective mechanisms (82).

Renal tubular epithelial cells depend heavily on mitochondrial oxidative phosphorylation and fatty acid oxidation for energy production (98, 99). Persistent lipotoxicity, inflammation, and oxidative stress may impair fatty acid oxidation, induce mitochondrial dysfunction, and promote compensatory glycolytic activation (100). These metabolic shifts may activate the NLRP3 inflammasome, transforming growth factor-β/Smad (TGF-β/Smad) signaling, and other profibrotic pathways (97), potentially facilitating progression from functional impairment to structural remodeling.

ART-related renal effects are mechanistically heterogeneous and may complicate the interpretation of CKM-related kidney vulnerability in treated PLWH. Tenofovir disoproxil fumarate (TDF) can accumulate in proximal tubular cells and cause mitochondrial injury, leading to tubular proteinuria, normoglycemic glycosuria, phosphate wasting, Fanconi-like tubulopathy, and declining kidney function; risk is greater with pre-existing kidney impairment or pharmacokinetic boosting (20, 94). Tenofovir alafenamide (TAF) results in lower systemic tenofovir exposure and generally has a more favorable renal safety profile (101). Atazanavir may cause crystal-related kidney injury through urinary precipitation and crystal deposition, resulting in nephrolithiasis or tubulointerstitial injury (94). By contrast, dolutegravir, bictegravir, cobicistat, and rilpivirine may increase serum creatinine by inhibiting tubular creatinine secretion without reducing true glomerular filtration (20). Distinguishing true ART-related kidney injury from transporter-mediated creatinine changes is therefore important to avoid misattributing renal abnormalities to residual HIV activity or CKM-related inflammatory and metabolic processes.

Residual viral expression may add another layer of renal stress through viral proteins such as Tat, Nef, and Vpr, particularly when combined with certain ART exposures, genetic susceptibility, and ongoing metabolic, inflammatory, or hemodynamic stressors (6, 94, 102).

### 6.4 Inter-organ feedback loops in multifactorial CKM-related tissue vulnerability

**Figure 3:**
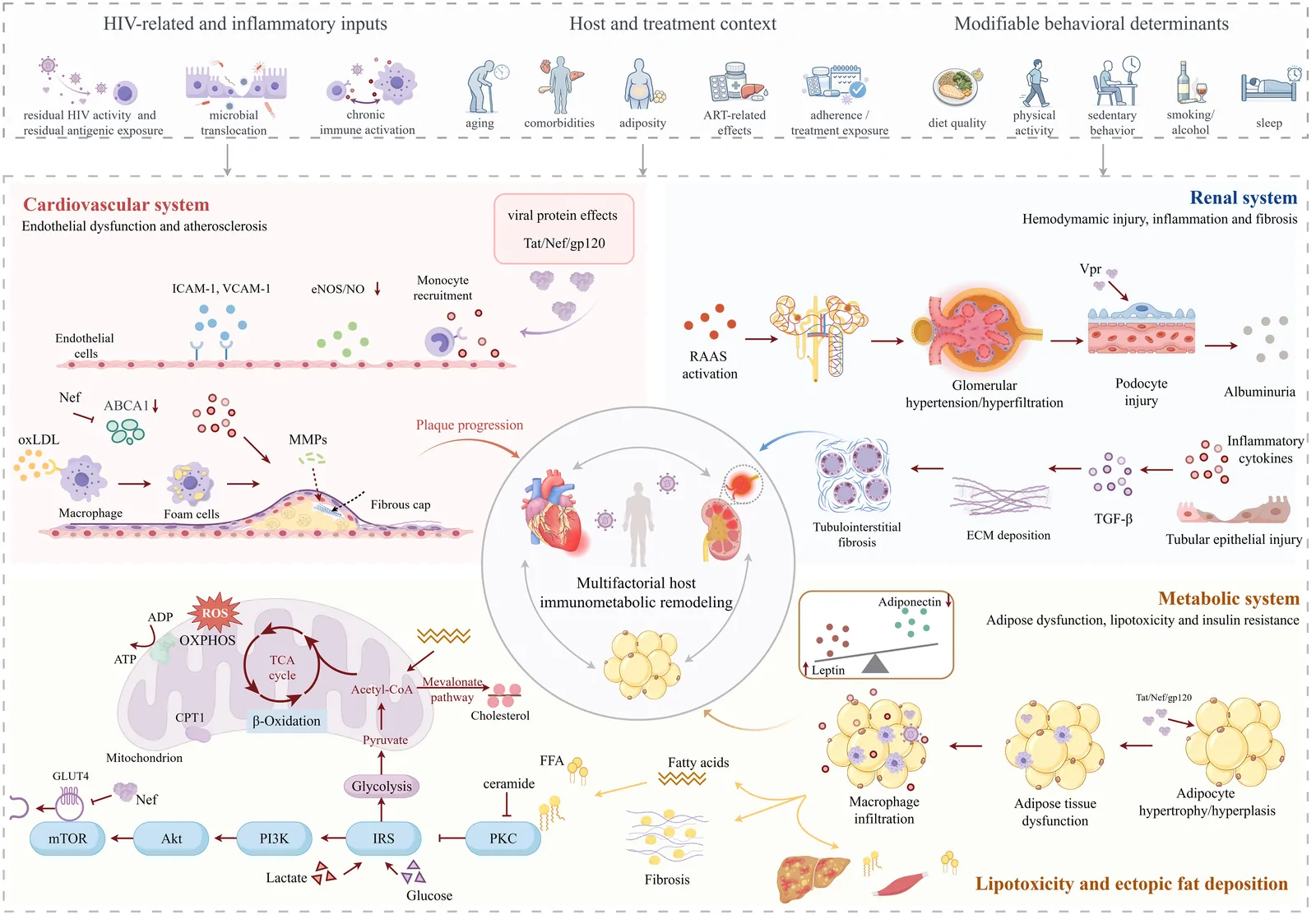
Reciprocal inter-organ interactions contributing to cardiovascular–kidney–metabolic vulnerability during treated HIV infection. The upper panel summarizes HIV-related and inflammatory inputs, host and treatment context, and modifiable behavioral determinants that may influence host phenotypes. The cardiovascular panel depicts endothelial dysfunction, monocyte recruitment, impaired cholesterol efflux, foam-cell formation, and atherosclerotic plaque remodeling. The renal panel shows glomerular hypertension, podocyte injury and albuminuria, together with tubular epithelial injury, inflammatory signaling, extracellular-matrix deposition, and tubulointerstitial fibrosis. The metabolic panel illustrates impaired insulin signaling, mitochondrial and lipid metabolic dysfunction, adipose-tissue inflammation, lipotoxicity, and ectopic fat deposition. The central circular arrows indicate reciprocal interactions among cardiovascular, kidney, and metabolic systems. Viral proteins are presented as potential additional stressors, not as exposures demonstrated in all individuals with LLV. The pathways are multifactorial and do not imply that LLV independently causes the illustrated organ injury. ABCA1, ATP-binding cassette transporter A1; ADP, adenosine diphosphate; Akt, protein kinase B; ATP, adenosine triphosphate; CPT1, carnitine palmitoyltransferase 1; ECM, extracellular matrix; eNOS, endothelial nitric oxide synthase; FFA, free fatty acids; GLUT4, glucose transporter 4; ICAM-1, intercellular adhesion molecule 1; IRS, insulin receptor substrate; MMPs, matrix metalloproteinases; NO, nitric oxide; OXPHOS, oxidative phosphorylation; oxLDL, oxidized low-density lipoprotein; PI3K, phosphoinositide 3-kinase; PKC, protein kinase C; RAAS, renin–angiotensin–aldosterone system; TCA, tricarboxylic acid cycle; TGF-β, transforming growth factor beta; VCAM-1, vascular cell adhesion molecule 1. Created by figdraw.com.

A defining feature of CKM-related risk is reciprocal feedback among metabolic, vascular, and kidney systems, rather than isolated organ injury (103). Adipose tissue inflammation and IR increase circulating free fatty acids, inflammatory cytokines, and lipotoxic mediators, thereby promoting endothelial dysfunction and vascular inflammation. Vascular injury and reduced compliance increase peripheral resistance and microcirculatory stress, whereas kidney dysfunction can impair clearance of inflammatory mediators, uremic toxins, and metabolic waste products, further amplifying oxidative stress, vascular calcification, and IR (95–97, 104). **Figure 3** summarizes these reciprocal inter-organ interactions and situates them within broader HIV-related, behavioral, adiposity-related, and treatment-related contexts. The contextual determinants relevant to interpreting LLV within this network are discussed in the following section.

## 7 Contextual determinants of LLV interpretation and CKM-related vulnerability

### 7.1 Behavioral and adiposity-related determinants

The interpretation of host phenotypes observed alongside LLV requires explicit consideration of the behavioral and metabolic context in which LLV is detected. Diet quality, physical activity, sedentary behavior, smoking, harmful alcohol use, sleep disturbance, and the extent and distribution of adiposity are relevant contextual factors when interpreting inflammatory, metabolic, and vascular phenotypes in PLWH (47, 50, 105, 106). Because these exposures frequently cluster and change over time, they should be assessed jointly and longitudinally, with their roles as independent determinants, confounders, mediators, or effect modifiers specified according to the causal question.

### 7.2 ART adherence and effective treatment exposure

ART exposure requires separate consideration because it may influence whether LLV occurs or is detected. Suboptimal adherence, treatment interruptions, drug–drug or drug–food interactions, and impaired drug absorption may reduce effective ART exposure and contribute to detectable viremia (9, 20). However, persistent or non-suppressible viremia may occur despite adequate adherence, including through viral production by clonally expanded infected-cell populations (23, 43). LLV should therefore be interpreted according to its magnitude and longitudinal pattern, together with adherence, treatment interruptions, pharmacologic exposure, potential drug interactions, and resistance history. Without such information, observed associations between LLV and host immunometabolic phenotypes may partly reflect incomplete treatment exposure and cannot be confidently attributed to biologically active residual HIV expression.

### 7.3 Regimen-specific metabolic effects and implications for interpretation

ART agents and regimen changes may have heterogeneous effects on body weight, adipose distribution, lipid and glucose metabolism, and other CKM-related risk profiles (47, 87, 107). These metabolic effects may shape CKM-related phenotypes even during sustained virologic suppression and should be distinguished from inadequate treatment exposure, which may contribute to detectable viremia. Effective ART remains foundational, and potential metabolic liabilities should not be interpreted as a rationale to compromise virologic suppression. Regimen selection or modification should instead be individualized by considering virologic efficacy, resistance profile, genetic barrier to resistance, adherence, tolerability, drug–drug and drug–food interactions, treatment history, metabolic profile, and comorbidity burden (9, 20, 21, 107). Accordingly, studies evaluating associations of LLV with immunometabolic or CKM outcomes should measure adherence, effective ART exposure, and regimen history longitudinally and avoid attributing observed risk to LLV without accounting for these treatment-related factors.

## 8 Positioning CKM-related risk within the context of treated HIV infection

Against this multifactorial background, the 2026 multisociety CKM guideline provides the reference framework for organizing established metabolic, kidney, and cardiovascular risk in PLWH (15). We apply the guideline-defined CKM staging without modification; LLV, residual HIV activity, and HIV-related biomarkers are considered contextual features rather than CKM staging variables.

The CKM staging construct spans Stages 0–4 according to progressively greater metabolic, kidney, and cardiovascular involvement: Stage 0 denotes the absence of CKM risk factors; Stage 1, excess or dysfunctional adiposity and/or prediabetes; Stage 2, metabolic risk factors and/or moderate- to high-risk CKD; Stage 3, subclinical CVD or risk equivalents, including very-high-risk CKD or a 10-year PREVENT-CVD risk ≥20%; and Stage 4, clinical CVD, with kidney failure distinguishing Stage 4b from Stage 4a (15). These stages reflect increasing pathophysiological burden and absolute cardiovascular risk rather than a deterministic sequence.

HIV/AIDS is recognized as a chronic inflammatory risk enhancer within the 2026 CKM guideline (15), supporting consideration of HIV-related context alongside conventional staging and quantitative risk assessment. Within this framework, persistent or recurrent LLV may serve as a pattern-dependent signal prompting further characterization of residual HIV activity, treatment exposure, and host immunometabolic recovery in selected ART-treated PLWH. Its potential translational value lies in whether LLV patterns or accompanying host signatures provide reproducible information beyond conventional CKM stage, PREVENT-estimated CVD risk, established risk enhancers, ART-related factors, and comorbidity burden (108).

**Figure 4:**
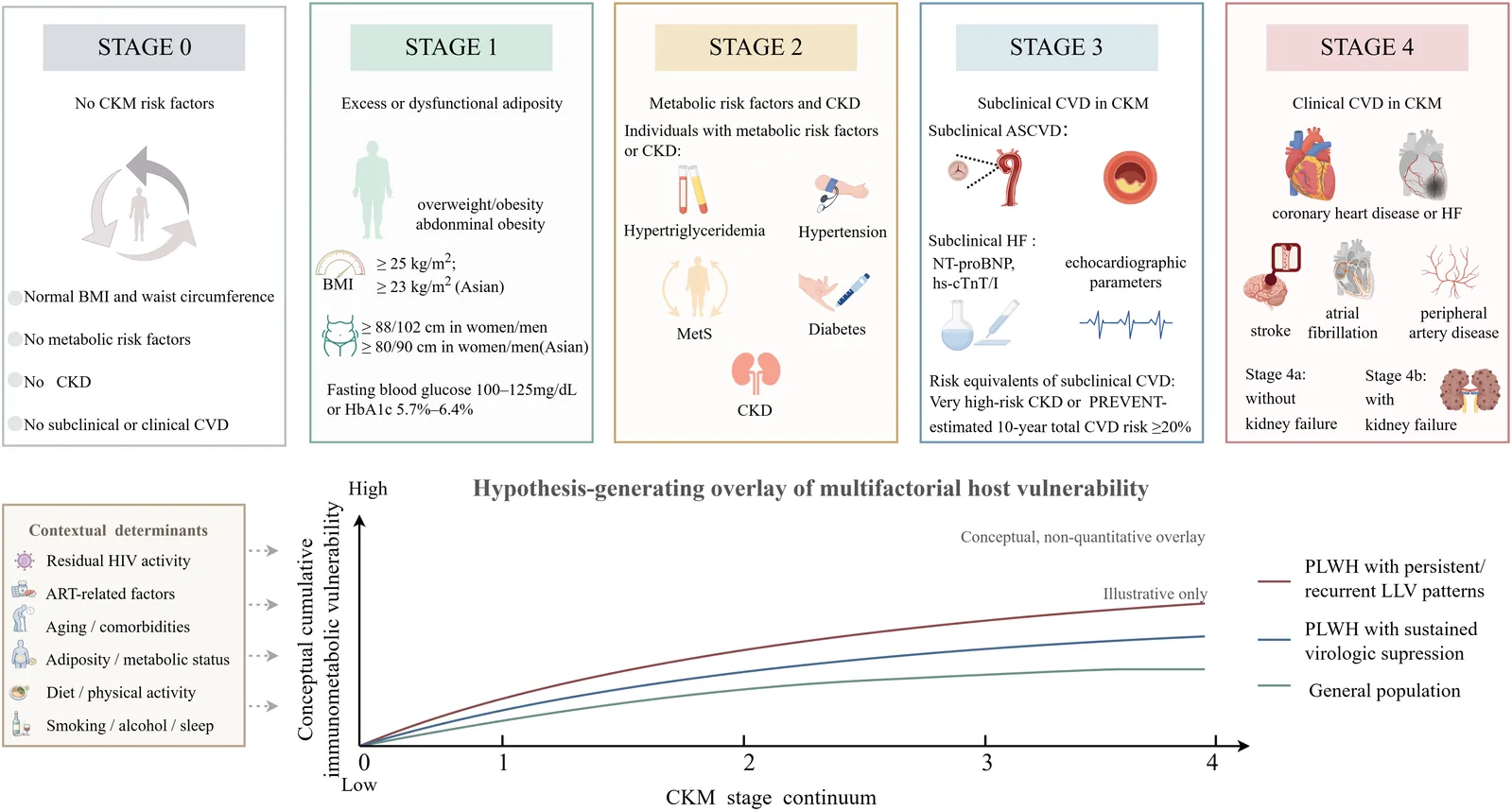
Conceptual integration of persistent or recurrent low-level viremia within the multifactorial cardiovascular–kidney–metabolic risk continuum. The upper panel summarizes the American Heart Association CKM stages, progressing from no apparent CKM risk factors at Stage 0, through excess or dysfunctional adiposity at Stage 1, metabolic risk factors or CKD at Stage 2, subclinical CVD or risk equivalents at Stage 3, and clinical CVD at Stage 4. The lower panel places residual HIV activity, ART-related factors, aging, comorbidities, adipose and metabolic status, and behavioral determinants alongside the formal CKM stages. The three curves represent hypothetical, non-quantitative trajectories of cumulative immunometabolic vulnerability in PLWH with persistent or recurrent LLV, PLWH with sustained virologic suppression, and the general population. Their shape, ordering, and separation are illustrative and have not been empirically validated. LLV is not part of CKM staging and should not be interpreted as an independent cause or determinant of CKM progression. ASCVD, atherosclerotic cardiovascular disease; BMI, body mass index; CKD, chronic kidney disease; CVD, cardiovascular disease; HbA1c, glycated hemoglobin A1c; HF, heart failure; hs-cTnT/I, high-sensitivity cardiac troponin T or I; MetS, metabolic syndrome; NT-proBNP, N-terminal pro-B-type natriuretic peptide. Created by figdraw.com.

Operationally, the proposed framework comprises five linked layers: (i) virologic characterization, to define the magnitude and longitudinal pattern of detectable HIV RNA; (ii) treatment and contextual assessment, incorporating ART adherence and exposure, regimen and resistance history, and relevant behavioral, adiposity-related, and metabolic determinants; (iii) host phenotyping, integrating immune, inflammatory, metabolic, vascular, and kidney-related responses; (iv) established CKM risk characterization, using guideline-defined CKM stage and PREVENT-CVD where appropriate, without incorporating LLV or HIV-related biomarkers into formal staging (15, 108); and (v) longitudinal reassessment, to determine whether LLV patterns or accompanying host signatures provide incremental information beyond conventional CKM stage and treatment-related determinants. This layered structure is intended for hypothesis generation and prospective validation rather than as a validated clinical risk score, biomarker panel, or LLV-specific management algorithm. Candidate measures for operationalizing the virologic, host-phenotyping, and CKM-related layers are detailed in Section 9.2 and **Table 2**, while **Figure 4** summarizes the conceptual positioning of LLV within multifactorial CKM risk.

**Table 2:** Candidate measures for contextualized longitudinal assessment of LLV patterns, host phenotypes, and CKM-related risk.

Domain	Core clinical measures	Selected additional measures	Main purpose
Virologic and treatment context	Serial plasma HIV RNA; LLV pattern; ART adherence and exposure; regimen, resistance, and treatment history	Cell-associated HIV RNA/DNA; measures of proviral intactness; viral sequencing	Contextualizes clinical LLV patterns relative to residual HIV activity and treatment exposure (8, 9, 20)
Immune recovery and dysfunction	CD4^+^ T-cell count; CD4^+^/CD8^+^ ratio	CD38^+^HLA-DR^+^ T-cell activation; PD-1; TIM-3/HAVCR2; senescence or TEMRA phenotypes	Identifies immune abnormalities not captured by conventional recovery markers (35, 61, 62, 66)
Inflammation and coagulation	hs-CRP where clinically indicated	IL-6; D-dimer; sCD14; sCD163	IL-6/hs-CRP: systemic inflammation; D-dimer: coagulation activation; sCD14/sCD163: monocyte–macrophage activation (113, 114)
Adiposity and metabolic risk	BMI; waist circumference; blood pressure; fasting glucose or HbA1c; triglycerides, HDL-C and LDL-C	Insulin resistance indices; adipokines; metabolomic and lipidomic profiles	Defines conventional CKM risk and potential immunometabolic context (15, 47, 83)
Kidney vulnerability	Serum creatinine/eGFR; UACR; urinalysis	Cystatin C; tubular protein markers; serum phosphate and urine glucose when TDF toxicity is suspected	Distinguishes glomerular, albuminuric and tubular injury and helps interpret ART-related effects (15, 94, 101)
Vascular and cardiac vulnerability	Clinical CVD history; PREVENT-CVD risk where appropriate	Carotid plaque; coronary artery calcium; echocardiography; hs-cTn or NT-proBNP	Characterizes subclinical or clinical CVD and refines conventional risk assessment (15, 108, 114–116)

Core clinical measures are routinely available for clinical characterization. Selected additional measures include clinically indicated or research-oriented assessments. These measures do not constitute a validated LLV-specific biomarker panel and should not be used independently to guide ART modification or CKM treatment. hs-cTn, high-sensitivity cardiac troponin; NT-proBNP, N-terminal pro-B-type natriuretic peptide.

## 9 Testable hypotheses and research priorities for residual HIV activity, host immunometabolic remodeling, and CKM-related risk

Building on the preceding sections, this section translates the proposed relationships among LLV, residual HIV activity, host immunometabolic remodeling, and CKM-related risk into testable research priorities. The framework should be tested rather than assumed, because LLV is a clinical monitoring signal rather than a direct surrogate for residual HIV activity, and CKM-related vulnerability is a longitudinal clinical trajectory rather than a predefined consequence of LLV. Future studies should address four linked questions: when and to what extent LLV corresponds to biologically active residual HIV expression; which host immunometabolic signatures remain associated with persistent or recurrent LLV after accounting for contextual determinants; whether these signatures precede, accompany, or improve prediction of CKM-related tissue vulnerability; and whether relevant virologic, inflammatory, metabolic, mitochondrial, lipid, and organ-protective pathways are therapeutically modifiable. The priorities operationalize the proposed framework as a linked translational pathway from biological interpretation of LLV, through contextualized host phenotyping and longitudinal CKM validation, to intervention testing.

### 9.1 When does LLV correspond to biologically active residual HIV expression?

The first priority is to determine when, and to what extent, LLV corresponds to biologically active residual HIV expression rather than assay variability, transient blips, or incomplete drug exposure related to adherence, treatment interruptions, drug interactions, or impaired absorption. Distinguishing isolated viral blips, persistent or recurrent LLV, and non-suppressible viremia is important for investigating whether detectable plasma HIV RNA reflects clonal release, intermittent proviral transcription, tissue-associated viral expression, or, in selected contexts, ongoing replication (9, 109). Addressing this question will require integrated virologic profiling that combines plasma HIV RNA with cell-associated HIV RNA and DNA, measures of proviral intactness, viral sequence dynamics, and tissue-reservoir assessments where feasible (110). Such approaches would clarify whether persistent or recurrent LLV corresponds to residual viral output, is accompanied by residual antigenic exposure, or remains primarily a nonspecific monitoring finding. This distinction is critical because LLV itself is a clinical monitoring signal, whereas residual HIV activity is the broader biological process more plausibly linked to continued host immune stimulation.

### 9.2 Which immunometabolic signatures accompany persistent or recurrent LLV after accounting for contextual determinants?

The next priority is to determine whether persistent or recurrent LLV is accompanied by reproducible host immunometabolic signatures after accounting for contextual determinants. Candidate measures for contextualized longitudinal assessment are summarized in **Table 2**. Core clinical characterization should include serial HIV RNA measurements, ART adherence and regimen history, conventional immune recovery indices, adiposity, blood pressure, glycemic and lipid measures, kidney function, albuminuria, clinical cardiovascular disease history, guideline-defined CKM stage, and PREVENT-CVD risk estimation where clinically appropriate (9, 15, 20).

Research-oriented phenotyping may additionally incorporate cell-associated HIV RNA or DNA, measures of proviral intactness, viral sequencing (8), T-cell activation and checkpoint-associated phenotypes, systemic inflammatory markers such as IL-6 and hs-CRP, D-dimer as a marker of coagulation, sCD14, sCD163 as markers of monocyte–macrophage activation, epigenetic features consistent with trained-immunity-like reprogramming, mitochondrial dysfunction, oxidative stress, and lipidomic or metabolomic profiles (89, 111, 112).

These signatures should not be interpreted as LLV-specific without careful measurement and analysis of relevant demographic and metabolic characteristics, adiposity, behavioral exposures, ART adherence and regimen history, coinfections, and comorbidities (9, 47, 50, 105–107). Their roles should be specified using prespecified causal models because the same factor may act as a confounder, mediator, or effect modifier depending on the research question and timing of assessment. Repeated measurement will be important, as a single baseline assessment may not adequately capture changes in adherence, ART exposure, adiposity, behavior, or comorbidity status over time. Integrated longitudinal analyses should therefore combine the multidomain measures summarized in **Table 2** rather than relying on single biomarkers. Such studies would help clarify whether the host profile associated with persistent or recurrent LLV reflects a reproducible residual HIV-associated state, a broader CKM-related risk phenotype, or an interaction between the two.

### 9.3 Do host immunometabolic signatures precede or improve prediction of CKM-related tissue vulnerability?

A third priority is to test whether host immunometabolic signatures precede, accompany, or improve prediction of CKM-related tissue vulnerability. Prospective cohorts should repeatedly assess the measures summarized in **Table 2** together with organ-specific outcomes to clarify temporal, cumulative, and potential dose–response relationships. Relevant outcomes include adipose dysfunction, insulin resistance, dyslipidemia, endothelial dysfunction, subclinical atherosclerosis, albuminuria, estimated glomerular filtration rate decline, and other early indicators of metabolic, vascular, and kidney vulnerability. Longer follow-up should examine changes in CKM stage, including progression or regression, together with hard clinical endpoints, such as cardiovascular events, kidney failure, and all-cause mortality. Higher CKM stages have been associated with greater risks of cardiovascular and all-cause mortality (117–119). Analyses should determine whether LLV patterns or accompanying host immunometabolic signatures provide incremental prognostic information beyond conventional CKM determinants, ART-related factors, and comorbidity burden.

Mechanistic studies should further test whether residual HIV activity contributes independently to host immunometabolic remodeling, interacts with pre-existing tissue stress, or merely accompanies broader CKM-related phenotypes. Single-cell profiling, tissue-based reservoir studies, viral protein detection, mitochondrial assays, lipidomics, and metabolic flux analyses may help distinguish direct viral or antigenic effects from parallel microbial, metabolic, behavioral, and treatment-related influences.

### 9.4 Can host immunometabolic states and CKM-related risk be modified in ART-treated PLWH?

Finally, intervention-oriented research should distinguish guideline-based virologic management from experimental host- and CKM-directed strategies (21). Studies should test separately whether interventions that alter virologic or antigenic exposure modify host immunometabolic signatures, and whether host- or CKM-directed interventions improve CKM-related outcomes independently of changes in LLV. Study designs should prespecify virologic, immunologic, metabolic, and organ-specific endpoints and stratify participants according to LLV pattern, direct measures of residual HIV activity, ART regimen and exposure, baseline host phenotype, and established CKM risk factors.

Behavioral intervention studies should examine whether improving dietary quality, increasing physical activity, reducing sedentary behavior, supporting smoking cessation, reducing harmful alcohol use, improving sleep, and reducing central or visceral adiposity modify inflammatory, metabolic, vascular, kidney, or CKM-related outcomes in ART-treated PLWH (50, 105). For dietary interventions specifically, studies should prespecify measures of diet quality and fiber intake, gut microbial ecology, microbial metabolites, mucosal barrier integrity, microbial translocation, and downstream immune or inflammatory phenotypes, and metabolic and CKM-related outcomes to test this proposed host-directed pathway. Any claim that dietary or other behavioral interventions affect LLV, residual HIV transcription, viral protein expression, or reservoir activity should require direct measurement and demonstration of the relevant virologic outcomes.

Established CKM-directed therapies, including statins, renin–angiotensin–aldosterone system inhibitors, sodium-glucose cotransporter 2 inhibitors, glucagon-like peptide-1 receptor agonists, and mineralocorticoid receptor antagonists, should be used according to guideline-based indications in appropriately selected PLWH (15, 115, 120–123). Future studies should determine whether their effects on host immunometabolic signatures or CKM-related outcomes vary according to LLV patterns or residual HIV activity; these therapies should not be framed as LLV-specific interventions.

## 10 Limitations of the evidence base and proposed framework

Interpretation of the evidence synthesized in this review is limited by substantial heterogeneity in how LLV is defined and measured. Viral-load thresholds, assay sensitivity, monitoring intervals, confirmation requirements, and criteria for persistence or recurrence vary across guidelines and research settings (9). Isolated viral blips, persistent or recurrent LLV, and non-suppressible viremia should therefore not be assumed to represent biologically equivalent states. Moreover, plasma HIV RNA alone cannot identify the source or biological significance of detectable viremia, including whether it reflects viral production from clonally expanded infected-cell populations, intermittent proviral transcription, tissue-associated viral expression, incomplete ART exposure, or ongoing cycles of replication (7, 28). This heterogeneity limits cross-study comparability and may bias associations with immune, metabolic, or clinical outcomes.

The evidence base is also constrained by the scarcity of longitudinal studies with repeated, temporally aligned virologic, immunologic, metabolic, and organ-specific measurements. Consequently, the temporal ordering among LLV, residual HIV activity, residual antigenic exposure, host immunometabolic phenotypes, and CKM-related outcomes remains uncertain, substantially limiting causal inference and mediation analysis. Direct evidence linking LLV to trained-immunity-like reprogramming, durable immunometabolic remodeling, or subsequent CKM progression is particularly sparse. Much of the mechanistic rationale is instead derived from studies of HIV persistence and viral proteins, experimental models, or related inflammatory and cardiometabolic disease settings. These findings support biological plausibility but do not establish LLV as an upstream cause or demonstrate mediation through the proposed host pathways.

Interpretation is further complicated by contextual determinants that may influence detectable viremia, host phenotypes, and CKM-related outcomes through different causal roles. Relevant factors include ART regimen and exposure, adherence and treatment history, demographic and metabolic characteristics, adiposity and comorbidities, coinfections, behavioral factors, and broader social determinants. Depending on the research question and timing of measurement, the same factor may act as an independent determinant, confounder, mediator, or effect modifier; adjustment based on a single baseline assessment may therefore be inadequate. The CKM framework also has clear boundaries. It was not developed as an HIV-specific or LLV-specific staging system (15), and the incremental value of adding LLV patterns or accompanying host signatures to established CKM determinants has not been demonstrated. Its use here is therefore organizing and hypothesis-generating rather than a validated causal, prognostic, or management model.

Finally, this narrative review integrates virologic, immunological, metabolic, and clinical evidence across several rapidly evolving research domains. Given the narrative approach, selective coverage and interpretive bias cannot be excluded. Direct and indirect evidence was necessarily synthesized across heterogeneous populations, assays, experimental systems, and clinical outcomes, limiting the precision of cross-domain inference. Emerging areas such as residual antigen detection, trained-immunity-related reprogramming, lifestyle determinants, and regimen-specific metabolic effects could not be addressed exhaustively.

## 11 Conclusions and future directions

Persistent or recurrent LLV should be interpreted as a pattern-dependent virologic signal rather than as a direct measure of residual HIV activity, residual antigenic exposure, or host immunometabolic remodeling. Its biological significance depends on magnitude and trajectory, treatment context, and whether accompanied by independent evidence of residual HIV activity or host immune perturbation.

The principal contribution of this review is an integrated, hypothesis-generating framework in which LLV is considered alongside direct measures of residual HIV activity, host immunometabolic phenotypes, contextual determinants, and established CKM risk factors. The CKM framework is used to organize interrelated metabolic, vascular, and kidney vulnerability without redefining CKM staging or positioning LLV as a cause of CKM syndrome. The central translational question is whether LLV patterns or accompanying host signatures provide clinically meaningful information beyond established treatment-related and CKM determinants.

From a clinical perspective, persistent or recurrent LLV, or rising viremia, should continue to prompt guideline-based virologic assessment, including repeat viral-load testing and evaluation of adherence, ART exposure, drug interactions, and resistance risk. The proposed framework does not justify automatic ART intensification or host-directed treatment solely on the basis of LLV. CKM prevention in PLWH should remain grounded in durable virologic suppression and individualized ART management, together with evidence-based control of blood pressure, lipids, glycemia, kidney risk, and adiposity. Dietary quality, regular physical activity, reduced sedentary time, smoking cessation, avoidance of harmful alcohol use, and attention to sleep should form part of this integrated approach, but these measures should not be interpreted as interventions proven to reduce LLV or residual HIV activity.

Future studies should establish when and to what extent LLV corresponds to biologically active residual HIV expression, identify reproducible host immunometabolic signatures after accounting for contextual determinants, and determine the temporal relationships of these signatures with CKM-related outcomes. Mechanistic and interventional research should further test whether modifying virologic or antigenic exposure alters host signatures and whether host- or CKM-directed interventions improve relevant outcomes independently of changes in LLV. Temporal ordering, incremental clinical value, and mechanistic mediation require prospective validation before the proposed framework can inform LLV-specific assessment or management.
